# Effects of Simulation Practicum Using Flipped Learning for Korean Nursing Students

**DOI:** 10.3390/ijerph17186829

**Published:** 2020-09-18

**Authors:** Minkyung Gu, Sohyune R. Sok

**Affiliations:** 1 Department of Nursing, College of Science and Technology, Daejin University, Pocheon-si, Gyeonggi-do 11159, Korea; g-minkyung@hanmail.net; 2 College of Nursing Science, Kyung Hee University, Seoul 02447, Korea

**Keywords:** simulation, flipped learning, nursing student

## Abstract

This study aimed to examine the effects of simulation practicum using flipped learning on nursing competency, core basic nursing skill (subcutaneous injection) performance, self-efficacy, and learning satisfaction of Korean nursing students. This study used a quasi-experimental pretest-posttest control group design. The samples used were from 101 nursing students (Intervention 1: *n* = 34; Intervention 2: *n* = 34; Control: *n* = 33) over 20 years old in G provinces, Korea. For the experimental interventions, participants in the intervention group 1 were applied by simulation practicum using flipped learning, and those in intervention group 2 were applied by simulation practicum. Participants in the control group were applied by lecture-based practicum using a model. The measures were the study participants’ general characteristics survey, the nursing competency scale, the core basic nursing skill (subcutaneous injection) performance scale, the self-efficacy scale, and the learning satisfaction scale. There were statistically significant differences in nursing competency, core basic nursing skill (subcutaneous injection) performance, self-efficacy, and the learning satisfaction among the three groups. It was seen in this study that simulation practicum using flipped learning was the most effective teaching and learning method for the nursing practicum of Korean nursing students. The simulation practicum using flipped learning can be useful in providing nursing practicum to nursing students.

## 1. Introduction

Our society is making various efforts to produce competent nurses for an improved quality of medical care and to ensure the safety of patients so that they can respond appropriately to the rapidly changing healthcare environment worldwide. In 2015, the Accreditation Act for Education in Nursing mandated passing the National Assembly, and based on this, the quality of nursing education is managed through an ongoing improvement program [1].

Currently, it is becoming difficult to acquire nursing knowledge and skills professionally because of the strengthening of privacy standards of patients in hospitals and out of respect for human rights [2]. The opportunities for nursing students to perform direct nursing during practical training are gradually decreasing, and observation-based practical training is predominantly conducted instead [2,3,4]. In this regard, Korean nursing colleges have raised the issue that such clinical training has a clear limitation in producing competent nurses with nursing performance ability. The teaching and learning method, using simulations, has been proposed as a way to overcome this [3,4,5].

The teaching and learning method, using simulations, allows learners to actively participate in learning in order to raise their nursing performance ability based on clinical field cases [6,7,8]. Further, simulation practicum contributes to the improvement of nursing students’ problem-solving ability, clinical performance, and knowledge, as well as some degree of learning satisfaction [6,7,9]. However, the teaching and learning method, using simulations, is known to increase stress because of the tension and anxiety of actual situations, and it is reported that it causes fears of making mistakes and decreases the self-efficacy and learning satisfaction of nursing students [5,6,10]. The use of simulations as a teaching and learning method has become part of the learning strategy, even though there are limitations to the problem-solving process and the development of the clinical practice application ability of nursing students. Therefore, a teaching and learning method, as a new approach, is deemed necessary to overcome the limitations and difficulties of the teaching and learning method using simulations and to further improve the quality of nursing education in an evolving healthcare environment [11,12,13].

Flipped learning is a reverse concept learning method that breaks the traditional instructional education method. Flipped learning is generally used in theoretical subject courses, and it improves the learning ability of students [12,14,15,16]. Flipped learning helps learners to solve performance tasks by providing core educational contents in advance through the instructional media designed by the instructors. Therefore, the strategy of the instructor in flipped learning is infinite and diverse, and the use of educational media is the most important feature [17]. In simulation practicum using flipped learning, self-directed learning, which consists of an online class video lecture, makes learners aware of different situations in advance [15,17]. It can cultivate the problem-solving process for learners to design and explore by becoming a team member through the simulation practicum. However, it can be said that cooperative learning between the instructor and the learner is very important in the offline class of flipped learning due to the learner’s limitation in deep learning progression with self-directed learning via online class video lectures [17,18]. In the offline class, the instructor corrects the errors found in the performance task of the learners so that they can perform error-free practical tasks [14,15]. In the current situation, where there is a lack of innovative teaching and learning methods to improve the quality of nursing education [17], flipped learning can be used to develop a simulation practicum for nursing students and to verify its effectiveness is significantly valuable for nursing pedagogy [18].

Based on Ahn and Kwon’s [19] for The Korean Association for Science Education ‘STEAM’ model, and Fitts’ [20], three stages of skill acquisition theory, the conceptual framework of this study. was designed by reviewing the literature and previous studies. The Korean Association for Science Education ‘STEAM’ model has the ability to solve problems in a creative and integrated manner by increasing the overall knowledge and understanding of various fields, and skill acquisition theory is designed to explain the changes that occur when learners acquire skills. In this study, self-directed pre-learning, which involves simulation practicum lectures using flipped learning, develops the problem-solving process so that the learners will be able to design and explore various situations by recognizing them in advance [9,19]. Eventually, the repetition and practice of this process will enable the learner to finally accomplish the task successfully and to improve his or her problem-solving ability, thereby enhancing their sense of achievement in learning [4,15,17,21].

This study will make a contribution the development of a teaching and learning method that can reduce the tension, anxiety, and stress of nursing students that may occur during nursing practicum, and to improve their learning satisfaction in order to apply it to the curriculum. The aims of this study were to examine the effects of the simulation practicum using flipped learning on nursing competency, core basic nursing skill (subcutaneous injection) performance, self-efficacy, and the learning satisfaction of Korean nursing students.

## 2. Material and Methods

### 2.1. StudyDesign and Participants

A quasi-experimental pretest–posttest control group design was employed. The study participants included 101 nursing students (Intervention 1: *n* = 34, Intervention 2: *n* = 34, Control: *n* = 33) in a nursing college in Korea. After obtaining the permission of the nursing college, an explanation of the research participation was posted on the bulletin board of the nursing college. The participants were included with convenience sampling into this study, and they were randomly assigned to each group. The participants were nursing students that were 20 years or older and agreed to take part in it. They completed the fundamental nursing subject in the second year of the nursing curriculum, and had no experience in clinical nursing practicum or flipped learning. All participants completed the study, and there was no retention. Sample size adequacy (*N* = 30 in each group) using three-groups *F*-test, G power 3 analysis software (Informer Technologies Inc., Los Angeles, CA, USA) was estimated based on an alpha level = 0.05, effect size = 0.25, and power = 0.80 [22]. Therefore, the sample size in the study was adequate.

### 2.2. Interventions

Intervention Group 1: Simulation Practicum using Flipped Learning

In the online class video lecture, which is a simulation practicum using flipped learning, the researcher produced a 10 to 15-min video on the core contents of priority nursing problems in nursing care for hyperglycemic patients. For the nursing students in intervention group 1, a video lecture link was delivered so that only they could access the online class service of the college of nursing. Prior to conducting the simulation practicum, the students were guided to watch the video lecture repeatedly anytime and anywhere. The researcher introduced effective learning strategies and clinical practice guides designed for nursing students, and provided immediate feedback in the offline class regarding the video lecture and simulation practicum to make them more understandable for the students. In the clinical field scenario for hyperglycemic patient nursing, a simulation practicum was conducted for 15 min for each group in the role of 1 charge nurse, 2–3 nurses, and 1 doctor. After the simulation practicum, the students wrote a personal reflection journal to review the overall content of the online and offline classes in the simulation practicum using flipped learning. In addition, they had a 20-min debriefing time for the entire group to discuss what they needed to learn more in order to properly deal with real situations.

Intervention Group 2: Simulation Practicum

In intervention group 2, the researcher explained the environmental and physical situations of nursing care for hyperglycemic patients by group for 10 to 15 min before starting the simulation practicum. Afterwards, a simulation practicum was conducted for 15 min for each group in the role of 1 charge nurse, 2–3 nurses, and 1 doctor. After the simulation practicum, the students wrote a personal reflection journal and had 20-min debriefing time for the entire group to discuss what kinds of problems the patients had and what they found to be the most parts of what they learned that day.

Control Group: Lecture-Based Practicum Using a Model

The control group consisted of a lecture-based practicum using a model. Here, the researcher explained the contents that were necessary for the nursing care of hyperglycemic patients. The lecture contents in the lecture-based practicum using a model consisted of the same contents as those used in the video lecture for intervention group 1. The nursing students alternated with each other for the practicum by group, and they had time to present and discuss the rationale regarding priority nursing problems for the patients.

### 2.3. Measures

A survey of the participants’ general characteristics was developed by researchers and it consisted of 4 items in total: gender, age, grade, and major satisfaction.

The nursing competency scale developed by Clark [23] was revised by Hur et al. [24] to Korean version. This scale included assess–reassess to clients (5 items), health history (5 items), critical thinking (5 items), results of diagnosis or tests (5 items), education for clients (5 items), and communication (5 items). The scale was used to measure the level of nursing competency of the participants. It consisted of a total of 30 questions using a 5-point Likert scale. The range of score was 30 to 150 points. The higher the score of the respondent, the higher the levels of nursing competency. Reliabilities in this study were Cronbach’s α = 0.91.

The core basic nursing skill (subcutaneous injection) performance scale was developed by Korean Accreditation Board of Nursing Education [25] for Korean nursing students. This scale was used to measure the level of core basic nursing skill (subcutaneous injection) performance of the participants. It consisted of a total of 31 questions using a 3-point Likert scale, and the range of score was 31 to 93 points. The higher the score of the respondent, the higher the level of core basic nursing skill (subcutaneous injection) performance. The reliability in this study was Cronbach’s α = 0.89.

The self-efficacy scale developed by Sherer et al. [26] was translated and retranslated by researchers to a Korean version. This scale was used to measure the degree of self-efficacy of the participants. This scale included general self-efficacy (17 items) and social self-efficacy (6 items). It consisted of a total of 23 questions using a 5-point Likert scale, and the range of score was 23 to 115 points. The higher the score of the respondent, the higher the degree of self-efficacy. The reliability in this study was Cronbach’s α = 0.89.

The learning satisfaction scale was developed by You and You [27]. This scale was used to measure the degree of learning satisfaction. It consisted of a total of 24 questions using a 5-point Likert scale, and the range of score was 24 to 120 points. The higher the score of the respondent, the higher the degree of learning satisfaction. The reliability of the scale in this study was Cronbach’s α = 0.88.

### 2.4. Procedures

The researcher and the two research assistants produced flipped learning video lectures for this study, and prepared and organized the simulation practicum articles to facilitate education. In order to carry out a consistent evaluation, the researcher and the two research assistants thoroughly understood the contents of the nursing competency measurement tool and subcutaneous injection procedures, which served as the core basic nursing skill performance evaluation items, and finalized the detailed guidelines under mutual agreement. In order to prevent bias in the study, the researcher and the two research assistants repeatedly reconfirmed the contents of the evaluation and checked for missing or incorrect information. The researcher and the two research assistants were the same in the three groups, and they had the same didactic skills as a coordinator that was certified for simulation practicum from the Korean Society for Simulation in Healthcare. The researcher and the two research assistants measured the nursing competency and core basic nursing skill (subcutaneous injection) performance before, immediately after, and 2 weeks after the experimental intervention. The self-report questionnaire measured the general characteristics and self-efficacy before the experimental intervention, and the self-efficacy and learning satisfaction immediately after and 2 weeks after the experimental intervention. Further, in order to prevent error in the experimental diffusion effect, the study was first conducted on the control group, followed by intervention group 2 and intervention group 1 with two-week intervals. The duration of the collected data was from November 2018 to February 2019.

### 2.5. Statistical Analysis

The collected data were analyzed using SPSS 23.0 (IBM, Armonk, NY, USA). The frequency, percentage, and descriptive statistics were analyzed for the participants’ general characteristics. Homogeneity among three groups in the participants’ general characteristics and study variables at the baseline was analyzed using an F test. The effects of simulation practicum using flipped learning were analyzed by repeated measures by ANOVA. A *p*-value of less than 0.05 was considered statistically significant.

### 2.6. Ethical Considerations

The Institutional Review Board of D University in Korea approved this study (IRB No. 1040656-201801-SB-02-13). Participants were informed that they voluntarily take part in this study and can withdraw from the study at any time. Participants were also informed of the confidentiality of the data. Researchers obtained completed written consent forms from the study participants.

## 3. Results

### 3.1. General Characteristics of the Study Participants and Homogeneity

General characteristics of the study participants and homogeneity are shown in Table 1 and Table 2. The majority of participants were women. As for the age, 21 years old was the most common. In regards to satisfaction, most of the participants responded “Moderate or Satisfied”. As for the general characteristics of participants among the three groups, as well as the study variables before the intervention, there were no group differences at the baseline of a statistical significance level of *p* < 0.05 (Table 1 and Table 2).

### 3.2. Effects of Simulation Practicum Using Flipped Learning at the Immediately after and Two Weeks after Intervention

The effects of simulation practicum using flipped learning immediately after and two weeks after intervention are presented in Table 3. Immediately after the intervention, there were statistically significant differences in nursing competency, core basic nursing skill (subcutaneous injection) performance, self-efficacy, and learning satisfaction between the groups. The nursing competency score of intervention group 1 was 116.35, the core basic nursing skill (subcutaneous injection) performance score of intervention group 1 was 88.47, the self-efficacy score of the intervention group 1 was 91.68, and the learning satisfaction score of the intervention group 1 was 100.79, which was higher than intervention group 2 and the control group (Table 3).

Two weeks after the intervention, there were statistically significant differences in nursing competency, core basic nursing skill (subcutaneous injection) performance, self-efficacy, and learning satisfaction between the groups. The nursing competency score of intervention group 1 was 111.94, the core basic nursing skill (subcutaneous injection) performance score of the intervention group 1 was 84.77, the self-efficacy score of the intervention group 1 was 87.53, and the learning satisfaction score of experimental group 1 was 99.15, which was higher than the intervention group 2 and the control group (Table 3).

### 3.3. Effects of Simulation Practicum Using Flipped Learning

Effects of simulation practicum using flipped learning are shown in Table 4. In the interaction over time, nursing competency, core basic nursing skill (subcutaneous injection) performance, self-efficacy, and learning satisfaction had statistically significant differences according to the group and the time of measurement, as well as their interaction (Table 4).

## 4. Discussion

In intervention group 1 with the simulation practicum using flipped learning, nursing competency, core basic nursing skill (subcutaneous injection) performance, self-efficacy, and learning satisfaction were significantly higher than those in experimental group 2 and the control group immediately after the experimental intervention. In addition, two weeks after the experimental intervention and the interaction over time by the group had not significantly reduced compared to the other groups. The simulation practicum of intervention group 2, which was frequently performed in nursing education to date, supports the study result of reporting that nursing competency, self-efficacy, and learning satisfaction in nursing students decreased due to an unfamiliar practicum room environment [9]. Further, in the case of the lecture-based practicum using a model for the control group, it is suggested that it may help nursing students acquire knowledge to the extent that they only maintain the knowledge rather than actually apply and act as a negative factor in raising the critical thinking ability that is necessary for them in the future [28]. Direct comparison is currently difficult to achieve because there are no studies in Korea that carried out simulation practicum using flipped learning. However, the study into the teaching and learning method using flipped learning by Hew and Lo [12] supports the study result that the class design for systematic prior learning of instructors can increase the interaction between the learners and instructors. Furthermore, it can have a positive effect on class participation. It also supports the study result that education using flipped learning helps students to maximize their learning ability by enabling them to understand the content of the class in advance [12,16,17,18]. This can be important in complementing the shortcomings of the current nursing theory education, and proving that simulation practicum using flipped learning is an effective educational intervention. Therefore, using flipped learning is important to reducing errors in nursing performance and improving nursing competency through clinical case study, core basic nursing skill (subcutaneous injection) performance, self-efficacy, and learning satisfaction [13,17].

In this study, intervention group 1, for which experimental intervention was applied, demonstrated an improvement in nursing learner’s memory in knowledge peaks. This is similar to the results obtained by Kim and Heo’s [29], that the learner’s memory in knowledge peaks when measured immediately, and short-term memory is internalized into long-term memory, and repetitive practice is essential to switching theoretical knowledge into skills [4]. Therefore, it is necessary to recognize the importance of repetitive re-education at an appropriate time in order to maintain nursing competency, core basic nursing skill (subcutaneous injection) performance, self-efficacy, and the learning satisfaction of the nursing students.

In nursing education, the biggest advantage of practicum representing the clinical field is capturing the interest of the nursing students [21]. In the teaching and learning method using flipped learning, the instructor needs to expand and provide activity-oriented classes to extend the development and knowledge of various video lecture media, while taking into account the interests of the nursing students [13,16,21,30,31]. In addition, if nursing skills are gradually completed by increasing the interaction between the instructor and the learner using flipped learning, they will be able to make a significant contribution to the improvement of professional skills for nursing performance.

Based on the results of this study, simulation practicum using flipped learning can be applied to the curriculum in order to improve nursing competency, core basic nursing skill (subcutaneous injection) performance, self-efficacy, and learning satisfaction of the nursing students. In the current nursing education, simulation practicum using flipped learning can provide a new direction for clinical case teaching and learning method, and it can be applied as an effective educational intervention. The results from this study may be beneficial to developing policies for the nursing curriculum. It will ultimately contribute to the development of competent nurses with expertise.

Since this study was conducted on students from a single nursing college, it is necessary to be careful when generalizing the study results, and there is a limitation in extending the interpretation to all nursing students. Therefore, it is necessary to repeat and expand the study in the future, while taking into consideration the sampling of the subjects.

## 5. Conclusions

In conclusion, it was confirmed that the simulation practicum using flipped learning had positive effects on nursing competency, core basic nursing skill (subcutaneous injection) performance, self-efficacy, and learning satisfaction. Therefore, simulation practicum using flipped learning can be implemented in order to improve the nursing performance ability of Korean nursing students. An experimental study should be attempted in the future in order to develop and verify a nursing flipped learning model by establishing specific procedures and strategies for simulation practicum using flipped learning. Above all, a systematic curriculum is deemed necessary in order to improve simulation practicum using flipped learning.

## Figures and Tables

**Table 1:** General characteristics of the study participants and homogeneity.

Characteristics	Intervention	Intervention	Control	F	*p*
	Group 1	Group 2	Group		
	(*N* = 34)	(*N* = 34)	(*N* = 33)		
	n (%)				
Gender					
Male	7 (20.6)	4 (11.8)	6 (18.2)	1.009 †	0.604
Female	27 (79.4)	30 (88.2)	27 (81.8)		
Age (years)					
21	19 (55.9)	23 (67.6)	20 (60.6)	16.604 †	0.313
22	8 (23.5)	7 (20.6)	13 (39.4)		
23	2 (5.9)	4 (11.8)	0 (0.0)		
24	2 (5.9)	0 (0.0)	0 (0.0)		
25	3 (8.8)	0 (0.0)	0 (0.0)		
Grade					
≤2.0–2.9	3 (8.8)	0 (0.0)	3 (9.1)	11.123 †	0.125
3.0–3.9	25 (73.6)	34 (100.0)	24 (72.7)		
≥4.0–4.5	6 (17.6)	0 (0.0)	6 (18.2)		
Major satisfaction					
Satisfied	23 (67.6)	24 (70.6)	13 (39.4)	12.064 †	0.289
Moderate	11 (32.4)	10 (29.4)	17 (51.5)		
Dissatisfied	0 (0.0)	0 (0.0)	3 (9.1)		

† Fisher exact test.

**Table 2:** Homogeneity of study variables at the pre-intervention among three groups.

Study Variables	Intervention	Intervention	Control	F	*p*
	Group 1	Group 2	Group		
	(*N* = 34)	(*N* = 34)	(*N* = 33)		
		Mean ± SD			
Nursing competency	59.44 ± 6.00	61.79 ± 5.13	60.15 ± 3.42	1.993	0.142
Core basic nursing skill	73.62 ± 4.57	75.12 ± 5.99	73.00 ± 5.09	1.448	0.240
performance					
(subcutaneous injection)					
Self-efficacy	52.85 ± 5.76	51.74 ± 4.10	54.33 ± 5.57	2.106	0.127

**Table 3:** Effects of simulation practicum using flipped learning at the immediately after and two weeks after experimental intervention.

Study Variables	Group	Post		Post 2 Weeks	
		Mean ± SD	F	Mean ± SD	F
			(*p*)		(*p*)
			Scheffe		Scheffe
Nursing competency	Intervention 1 ^a^	116.35 ± 1.87	2155.139	111.94 ± 2.69	873.818
	(*N* = 34)				
	Intervention 2 ^b^	96.29 ± 2.67	(<0.001 *)	84.77 ± 8.26	(<0.001 *)
	(*N* = 34)				
	Control ^c^	59.76 ± 5.30	a > b > c	55.42 ± 3.99	a > b > c
	(*N* = 33)				
Core basic nursing skill performance (subcutaneous injection)	Intervention 1 ^a^	88.47 ± 1.73	43.534	84.77 ± 2.71	134.708
	(*N* = 34)				
	Intervention 2 ^b^	82.82 ± 2.04	(<0.001 *)	71.41 ± 2.80	(<0.001 *)
	(*N* = 34)				
	Control ^c^	83.58 ± 3.88	a > b >c	73.03 ± 5.03	a > b > c
	(*N* = 33)				
Self-efficacy	Intervention 1 ^a^	91.68 ± 4.31	601.292	87.53 ± 4.32	621.770
	(*N* = 34)				
	Intervention 2 ^b^	81.09 ± 2.17	(<0.001 *)	61.82 ± 3.60	(<0.001 *)
	(*N* = 34)				
	Control ^c^	57.94 ± 5.17	a > b > c	52.15 ± 4.78	a > b > c
	(*N* = 33)				
Learning satisfaction	Intervention 1 ^a^	100.79 ± 3.22	519.480	99.15 ± 3.18	316.134
	(*N* = 34)				
	Intervention 2 ^b^	83.94 ± 1.87	(<0.001 *)	79.06 ± 5.81	(<0.001 *)
	(*N* = 34)				
	Control ^c^	64.64 ± 7.09	a > b > c	54.73 ± 10.73	a > b > c
	(*N* = 33)				

* *p* < 0.05. ^a, b, c^ Scheffe post hoc test.

**Table 4:** Effects of simulation practicum using flipped learning.

Variable	Group	Pre	Post	Post	Source	F *p*	Scheffe
				2 Weeks			
		Mean ± SD					
Nursing competency	Intervention 1 ^a^	59.44	116.35	111.94	Group	73,173.443	a > b > c
	(*N* = 34)	± 6.00	± 1.87	± 2.69		<0.001 *	
	Intervention 2 ^b^	61.79	96.29	84.77	Time	1382.410	
	(*N* = 34)	± 5.13	± 2.67	± 8.26		<0.001 *	
	Control ^c^	60.15	59.76	55.42	Group x Time	68.975	
	(*N* = 33)	± 3.42	± 5.30	± 3.99		<0.001 *	
Core basic nursing skills performance (subcutaneous injection)	Intervention 1 ^a^	73.62	88.47	84.77	Group	70,426.179	a > b > c
	(*N* = 34)	± 4.57	± 1.73	± 2.71		0.001 *	
	Intervention 2 ^b^	75.12	82.82	71.41	Time	515.499	
	(*N* = 34)	± 5.99	± 2.04	± 2.80		<0.001 *	
	Control ^c^	73.00	83.58	73.03	Group x Time	24.605	
	(*N* = 33)	± 5.09	± 3.88	± 5.03		<0.001 *	
Self-efficacy	Intervention 1 ^a^	52.85	91.68	87.53	Group	33,871.162	a > b > c
	(*N* = 34)	± 5.76	± 4.31	± 4.32		<0.001 *	
	Intervention 2 ^b^	51.74	81.09	61.82	Time	1162.369	
	(*N* = 34)	± 4.10	± 2.17	± 3.60		<0.001 *	
	Control ^c^	54.33	57.94	52.15	Group x Time	242.857	
	(*N* = 33)	± 5.57	± 5.17	± 4.78		<0.001 *	
Learning satisfaction	Intervention 1 ^a^	-	100.79	99.15	Group	22,756.969	a > b > c
	(*N* = 34)		± 3.22	± 3.18		<0.001 *	
	Intervention 2 ^b^	-	83.94	79.06	Time	94.027	
	(*N* = 34)		± 1.87	± 5.81		<0.001 *	
	Control ^c^	-	64.64	54.73	Group x Time	7.969	
	(*N* = 33)		± 7.09	± 10.73		<0.001 *	

* *p* < 0.05. ^a, b, c^ Scheffe post hoc test. x Interaction between group and time.
